# Detection and quantitation of HPV in genital and oral tissues and fluids by real time PCR

**DOI:** 10.1186/1743-422X-7-194

**Published:** 2010-08-19

**Authors:** William T Seaman, Elizabeth Andrews, Marion Couch, Erna M Kojic, Susan Cu-Uvin, Joel Palefsky, Allison M Deal, Jennifer Webster-Cyriaque

**Affiliations:** 1 Lineberger Comprehensive Cancer Center, University of North Carolina at Chapel Hill, NC, USA; 2 Department of Dental Ecology, School of Dentistry University of North Carolina at Chapel Hill, NC, USA; 3 Department of Microbiology and Immunology, University of North Carolina at Chapel Hill, NC, USA; 4 Biostatistics Core Facility, University of North Carolina at Chapel Hill, NC, USA; 5 College of Dental Medicine, Western University of Health Sciences, Pomona, CA, USA; 6 Department of Medicine, The Miriam Hospital, Brown University, Providence, RI, USA; 7 Department of Medicine, University of California, San Francisco, San Francisco, CA, USA

## Abstract

**Background:**

Human papillomaviruses (HPVs) remain a serious world health problem due to their association with anogenital/oral cancers and warts. While over 100 HPV types have been identified, a subset is associated with malignancy. HPV16 and 18 are the most prevalent oncogenic types, while HPV6 and 11 are most commonly responsible for anogenital warts. While other quantitative PCR (qPCR) assays detect oncogenic HPV, there is no single tube assay distinguishing the most frequent oncogenic types and the most common types found in warts.

**Results:**

A Sybr Green-based qPCR assay was developed utilizing degenerate primers to the highly conserved HPV E1 theoretically detecting any HPV type. A single tube multiplex qPCR assay was also developed using type-specific primer pairs and TaqMan probes that allowed for detection and quantitation of HPV6,11,16,18. Each HPV type was detected over a range from 2 × 10^1 ^to 2 × 10^6^copies/reaction providing a reliable method of quantitating type-specific HPV in 140 anogenital/cutaneous/oral benign and malignant specimens. 35 oncogenic and low risk alpha genus HPV types were detected. Concordance was detected in previously typed specimens. Comparisons to the gold standard detected an overall sensitivity of 89% (95% CI: 77% - 96%) and specificity of 90% (95%CI: 52% - 98%).

**Conclusion:**

There was good agreement between the ability of the qPCR assays described here to identify HPV types in malignancies previously typed using standard methods. These novel qPCR assays will allow rapid detection and quantitation of HPVs to assess their role in viral pathogenesis.

## Background

Papillomaviridae comprise a diverse family of non-enveloped, small circular double-stranded DNA viruses, capable of infecting mammals and birds [1]. In humans these viruses cause pathologies that range from benign warts to malignant cancer. It is now accepted that HPV is the causative agent of more than 90% of all cervical cancers [2]. HPV has also been found to be associated with anal cancer [3] and anogenital warts [4]. There is increasing evidence that HPV is associated with head and neck squamous cell carcinoma unrelated to smoking and/or alcohol consumption [5-7].

HPV types are divided into low- and high-risk groups with regards to their association with malignancy [8]. Low-risk HPV types 6 and 11 are most commonly detected in genital and anal warts, representing 90% of these cases [4]. Oncogenic HPV types 16 and 18 account for 70% of HPV-related cervical cancers. An increase in HPV viral loads has been correlated with disease progression in cervical cancer [9]. Similarly, elevated HPV viral loads were detected in HPV16-associated oropharyngeal squamous cell carcinomas [10]. These aforementioned associations highlight the importance of detecting, distinguishing, and quantitating both low risk and oncogenic HPV infections for monitoring and treating disease development and progression [9,10].

PCR represents a sensitive method for the detection of HPV DNA. Currently, standard nested HPV PCR can be performed using degenerative primers followed by direct sequencing of the PCR product. Alternatively, PCR products can be hybridized to DNA of known HPV types, to determine the type of HPV present in the PCR-amplified sample (e.g. Roche HPV Amplicor system). Both assays are time consuming and do not allow for the quantitation of viral DNA to address the role of viral load in disease progression.

Real time quantitative PCR (qPCR) allows for quantitation of DNA over 8 orders of magnitude [11]. While qPCR assays have been developed for HPV16 and/or 18 [12-16] relatively little has been done to develop assays that are capable of detecting other HPV types. Recently, two qPCR assays have been described that have increased the types of HPV that can be quantitatively detected [17,18]. In one assay, molecular beacon probes were used to distinguish low risk and oncogenic HPV types in a single multiplex reaction [18] although it did not allow for the specific determination of the HPV type. In another assay, TaqMan probes were designed to detect HPV16, HPV31, HPV 18/45 or HPV33/53/58/67 [17] in two separate multiplex reactions, did not allow the distinction between HPV33, 52, 58 or 67 and was unable to differentiate HPV18 from 45. More recently, a multiplex qPCR assay has been described that can quantitatively detect 7 oncogenic HPV types [19]. The assay is comprised of 2 reactions that detect only oncogenic HPV and no low risk HPVs. Thus, a single tube, multiplex reaction that can recognize common low and oncogenic HPV types involved in disease would be advantageous to the monitoring and detection of HPV infection. This would minimize reagents as well as decrease the chance for error that is inherent in performing multiple single or multiplex reactions.

This report describes two real time qPCR assays that can be used in tandem for detection of both common and uncommon HPV infection. Many distinct HPV types have been associated with disease hence, the first qPCR assay is a degenerate assay that targets the HPV E1 region of known HPVs and should allow for detection of any of these types. The second qPCR assay targets the coding region of the hypervariable loop V of the L1 gene of HPV6, 11, 16 and 18. These HPV types are associated with the majority of HPV related disease and are represented in the currently available quadrivalent vaccine. The primers and probes used in the development of the assay were designed to target and differentiate between these HPV types. In initial studies the vaccine has proven effective for prophylaxis against the initial infection by these types of HPV [20,21]. Vaccine efficacy has been assessed by either cytology and/or qualitative PCR. The qPCR assays described in this study will provide a more efficient quantitative means of monitoring of HPV types in populations vulnerable to HPV-associated disease.

## Materials and methods

### Subjects

This study was approved by the School of Medicine, Institutional Review Board University of North Carolina, Chapel Hill (IRB# 05-DENT-1263-ORC). Study subjects were identified through the UNC Healthcare Cancer Registry of >22,000 cases (1995-present). This Registry maintains a database of all patients diagnosed and/or treated for malignant neoplasm's at UNC Healthcare. Cases were subjects with a histologically-diagnosed cancer (confirmed by two independent pathologists). Well-characterized HPV positive controls from skin and anogenital lesions were provided by Joel Palefsky. For gynecological samples, cells were obtained after cervical lavage from HIV-positive subjects (IRB# 2080-05). Controls were chosen based on biopsies histologically-confirmed as benign. Paraffin embedded benign control tissues were defined as those not demonstrating any properties associated with malignancy (i.e. mitotic figures, hyperchromasia, pleomorphism and increased nuclear/cytoplasmic ratio).

### Isolation of cellular genomic DNA

CaSki and HeLa cell genomic DNA was obtained from Advanced Biotechnologies Inc. SiHA and DG-75 cellular DNA was isolated from cells using a Qiagen DNeasy Kit and used as positive controls in the real time PCR assay. Paraffin-embedded patient tissue was deparaffinized with xylene. Tissue was washed twice with 100% ethanol and dried. DNA was isolated using a Qiagen DNeasy kit according to the manufacturer's instructions.

### Cloning of HPV16 and 18 L1 amplicon

The 136 bp HPV16 L1 amplicon (6605-6741) was PCR-amplified from CaSki cell genomic DNA using Taq polymerase and standard PCR conditions. The 120 bp HPV18 L1 amplicon (6587-6707) was PCR-amplified from HeLa cell genomic DNA using Taq polymerase and standard PCR conditions. Both HPV16 L1 and HPV18 L1 amplicons were TA-cloned into pCR2.1-topo vector (Invitrogen) according to the manufacturer's instructions to obtain the plasmids, pHPV16L1 and pHPV18L1, respectively. Purified plasmids were sequenced to verify that the correct sequence was present and used to derive standard curves in the real time PCR assay for the detection of HPV16 and 18 L1 amplicons. Full-length HPV16 genome was PCR-amplified from Caski cell DNA using a Roche Expand Long Template PCR System and the primers HPV16BamHIF (5'-CCC**GGATCC**CCATGTACCAATGTTGCA-3') and HPV16BamHIR (5'-CCC**GGATCC**TTTGCCCCAGTGTTCC-3'). The 7.9 kb PCR fragment was TA-cloned into pCR2.1-topo vector to generate pHPV16. The plasmids pHPV6 and pHPV11 containing the entire genome of HPV6 and HPV11, respectively, were obtained from the American Type Culture Collection (ATCC) and were used to derive standard curves for HPV6 and 11 in the real time PCR assay.

### Sybr Green Real Time PCR

Real time PCR reactions that target the E1 region of HPV were performed using Roche Lightcycler Sybr Green master mix. Each reaction consisted of 1X Roche Lightcycler Sybr Green master mix, HPVE1F and HPVE1R (Table 1) in a 10 μl reaction. Regions of high homology between different types of HPV were identified with Vector NTI software (Invitrogen) and used to design degenerative "broad spectrum" HPV primers. Thermal cycle conditions consisted of an initial denaturation incubation at 95°C for 10 minutes followed by 50 cycles of alternating 95°C incubations for 10 seconds, 50°C incubations for 10 seconds and 72°C incubations for 30 seconds. Fluorescence was detected after every 72°C extension incubation. For standard curves, real time PCR was performed on a 10-fold dilution series of purified plasmids, pHPV6, pHPV11 and pHPV16, ranging from 2 × 10^1 ^to 2 × 10^6 ^copies/reaction.

**Table 1:** Sequence of HPV type-specific L1 primers and probes used for qPCR.

Oligonucleotide	Sequence (5'→3')	Concentration/Rx
HPV6L1F	TGGGGTAATCAACTGTTTGTTACTGTGGTA	400 nM

HPV6L1R	GCATGTACTCTTTATAATCAGAATTGGTGTATGTG	400 nM

HPV6L1probe	**Cy5-**GACATTATGTGCATCCGTAACTAC**-BHQ2**	200 nM

HPV11L1F	CTGGGGAAACCACTTGTTTGTTACTGTG	400 nM

HPV11L1R	CGCATGTATTCCTTATAATCTGAATTAGTGTATGTA	400 nM

HPV11L1probe	**TexasRed-**GACACTATGTGCATCTGTGTCTAA**-BHQ1**	800 nM

HPV16L1F	TTGTTGGGGTAACCAACTATTTGTTACTGTT	400 nM

HPV16L1R	CCTCCCCATGTCTGAGGTACTCCTTAAAG	400 nM

HPV16L1probe	**6FAM-**GTCATTATGTGCTGCCATATCTACTTC**-TAMRA**	400 nM

HP18L1F	GCATAATCAATTATTTGTTACTGTGGTAGATACCACT	400 nM

HP18L1R	GCTATACTGCTTAAATTTGGTAGCATCATATTGC	400 nM

HPV18L1probe	**HEX-**AACAATATGTGCTTCTACACAGTCTCCTGT**-BHQ2**	100 nM

HPVE1F1	ANANGCTGTGCAKGNNCTAAAACGAAG	300 nM

HPVE1R1	AGTTTCCACTTCAGTATTGCCATA	300 nM

HAPBF	TGAAGGTGGAGGACATTCCTCTA	400 nM

HAPBR	CTGGAATTGCGATTTCTGGTAA	400 nM

HAPBprobe	**Cyan500-**CGAGAATCACCCTGCCAGACTTCCGT**-BBQ**	100 nM

### Sequencing of PCR products

To sequence products generated by the Sybr-green-based qPCR assay, 3 microliters of ExoSAP-IT (USB Corporation) was added to 7 microliters of positive reactions. Reactions were incubated at 37°C for 15 minutes followed by incubation at 80° for 15 minutes. The entire volume was used for DNA sequencing using HPVE1F as a sequencing primer (Eton Bioscience Inc.). Blast sequence analysis was performed on generated sequences to identify homologies with other known HPV DNA.

### TaqMan Real time PCR reaction

Real time PCR reactions were performed using Roche Lightcycler TaqMan master mix. Each reaction consisted of 1X Roche Lightcycler TaqMan master mix, HPV specific primer pairs and fluorescently-tagged probes for types 6, 11, 16 and 18 in a total reaction volume of 10 μl (Table 1). The human ApoB (HAPB) gene is detected at a single copy in normal cells. Primers and probe targeting the cellular apoB gene as described by Sanchez and Storch [22] were included in the reactions to assess DNA integrity. Reactions were performed using a Roche Lightcycler 480 thermal cycler utilizing a 384 well block. Thermal cycle conditions consisted of an initial denaturation incubation at 95°C for 10 minutes followed by 40 cycles of alternating 95°C incubations for 15 seconds and 60°C incubations for 30 seconds. Fluorescence was detected after every 60°C extension incubation. For standard curves, real time PCR was performed on a 10-fold dilution series of each purified plasmid containing a type-specific L1 amplicon ranging from 2 × 10^1 ^to 2 × 10^6 ^copies/reaction. Quantitation of PCR products was performed using Roche Lightcycler 480 software. Color compensation was turned on in all assays to subtract bleed through between adjacent channels used to detect specific fluorescent tags.

### Statistical Analysis

Sensitivity and specificity were calculated as the proportion of true positives and true negatives, respectively, as found by the E1 broad spectrum/HPV 6,11,16,18 multiplex assays and exact 95% confidence intervals are provided.

## Results

### HPV EI Degenerate qPCR

**Figure 1:**
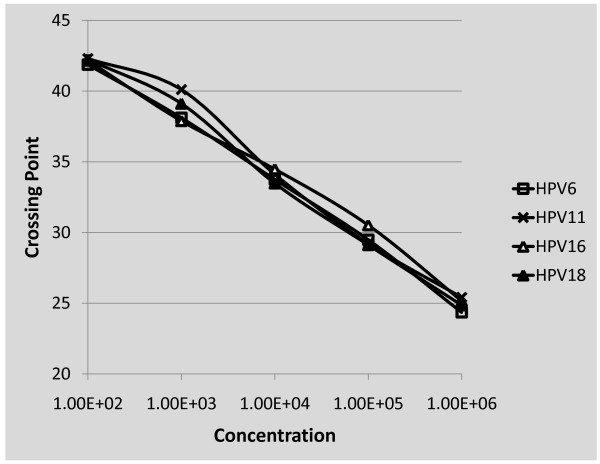
**Degenerate "broad spectrum" HPV E1 primers detect HPV6, 11, 16 and 18**. The E1 primer pair was used in a sybr green-based qPCR assay to determine the performance of this primer pair to detect and quantitate HPV. Reactions were performed using genomic clones of HPV6, 11, 16 or 18 as a standard curve that ranged from 1 × 10^6 ^to 1 × 10^2 ^copies and linear detection and quantitation of each amplicon was observed.

In order to efficiently detect HPV types present in clinical samples a broad spectrum E1 targeted Sybr green-based degenerate assay was developed. Two primers were selected from a highly conserved region of E1 region. To test the ability of these primers to detect HPV DNA, they were utilized in a qPCR assay using pHPV6, pHPV11, pHPV16 or pHPV18 plasmid DNA as a template (Figure 1). A range of 10^6 ^to 10^1 ^copies/reaction of each full-length HPV genome was used in the Sybr green qPCR assay. There was a linear relationship between the Ct values and cycle numbers for each of the plasmids when a range of between 1 × 10^2 ^copies and 1 × 10^6 ^copies of plasmid was present in the reaction, indicating that the assay was quantitative and had a lower limit of detection of 100 copies/reaction (Figure 1). Additionally, each standard had a similar curve indicating that all plasmids were amplified with equal efficiency regardless of the HPV type. This indicates that the degenerative Sybr Green-based qPCR assay is a reliable method for quantitating different types of HPV and should prove instrumental as an adjunct assay for quantitation of HPV types not detected by the multiplex qPCR assay (described in the following section). Following, the assays are utilized in 140 specimen types that reflect the pathogenesis of HPV in cutaneous and mucosal benign and malignant disease. We have assayed 39 anogenital/cutaneous specimens, 25 cervical lavages from HIV positive women, 54 oral cancers from smoker drinkers and 22 from non smoker non drinkers (Table 2).

**Table 2:** Summary of patient tissues and fluids used for the detection of HPV.

Sample	Sample origin	HPV (previously typed)	HPV E1 qPCR	HPV L1 qPCR
962046	anal virapap swab	11	+	11

950450	anal virapap swab	16	+	16

972696	anal virapap swab	33‡	+	-

971380	anal virapap swab	35‡	+	-

000699	anal virapap swab	40*	+	-

061675	anal virapap swab	45§	+	-

983165	anal virapap swab	52‡	+	-

972762	anal virapap swab	54	+	-

972666	anal virapap swab	59§	+	-

980280	anal virapap swab	69	+	-

980520	anal virapap swab	73	+	-

950474	anal virapap swab	83	+	-

081673	anal virapap swab	89	+	-

950472	anal virapap swab	16,53	+	16

950508	anal virapap swab	56,68§,84	+	-

950504	anal virapap swab	6,11,18,26,33,45§,54,73	+	6,11,18

950496	anal virapap swab	6,16,45§,53,58‡,61,70§,83,84	+	6

071455	anal preservecyt swab	42	+	-

071537	anal preservecyt swab	51	+	-

071113	anal preservecyt swab	71	+	-

071424	anal preservecyt swab	81	+	-

963601	Cervical lavage	67‡	+	-

950475	cervical virapap swab	6	+	6

950505	cervical virapap swab	18	+	18

950507	cervical virapap swab	26	+	-

962678	cervical virapap swab	31‡	+	-

950433	cervical virapap swab	32	+	-

962047	cervical virapap swab	53	+	-

950479	cervical virapap swab	54	+	-

972833	cervical virapap swab	55*	+	-

962721	cervical virapap swab	66	+	-

972835	cervical virapap swab	82	+	-

971371	cervical virapap swab	84	+	-

036465	cervical virapap cytobrush	58‡	+	-

040670	cervical preservecyt swab	56	+	-

CL1	Cervical lavage	N/D	-	-

CL2	Cervical lavage	N/D	+	6/16

CL3	Cervical lavage	N/D	+	-

CL4	Cervical lavage	N/D	-	-

CL5	Cervical lavage	N/D	-	-

CL6	Cervical lavage	N/D	+	6

CL7	Cervical lavage	N/D	+	6,18

CL8	Cervical lavage	N/D	-	-

CL9	Cervical lavage	N/D	-	-

CL10	Cervical lavage	N/D	-	-

CL11	Cervical lavage	N/D	+	-

CL12	Cervical lavage	N/D	-	-

CL13	Cervical lavage	N/D	-	-

CL14	Cervical lavage	N/D	-	-

CL15	Cervical lavage	N/D	-	-

CL16	Cervical lavage	N/D	-	-

CL17	Cervical lavage	N/D	-	-

CL18	Cervical lavage	N/D	+	-

CL19	Cervical lavage	N/D	-	-

CL20	Cervical lavage	N/D	-	-

CL21	Cervical lavage	N/D	+	6

CL22	Cervical lavage	N/D	-	-

CL23	Cervical lavage	N/D	+	-

CL24	Cervical lavage	N/D	-	-

CL25	Cervical lavage	N/D	-	-

A1	S/D Oral biopsy	N/D	-	-

A2	S/D Oral biopsy	N/D	-	-

A3	S/D Oral biopsy	N/D	-	-

A4	S/D Oral biopsy	N/D	-	-

A5	S/D Oral biopsy	N/D	-	-

A6	S/D Oral biopsy	N/D	-	-

A7	S/D Oral biopsy	N/D	-	-

A8	S/D Oral biopsy	N/D	-	-

A9	S/D Oral biopsy	N/D	-	-

A10	S/D Oral biopsy	N/D	-	-

A11	S/D Oral biopsy	N/D	-	-

A12	S/D Oral biopsy	N/D	-	-

B1	S/D Oral biopsy	N/D	-	-

B2	S/D Oral biopsy	N/D	-	-

B3	S/D Oral biopsy	N/D	-	-

B4	S/D Oral biopsy	N/D	-	-

B5	S/D Oral biopsy	N/D	-	-

B6	S/D Oral biopsy	N/D	-	-

B7	S/D Oral biopsy	N/D	-	-

B8	S/D Oral biopsy	N/D	-	-

B9	S/D Oral biopsy	N/D	-	-

B10	S/D Oral biopsy	N/D	-	-

B11	S/D Oral biopsy	N/D	-	-

B12	S/D Oral biopsy	N/D	+	16

C1	S/D Oral biopsy	N/D	-	-

C2	S/D Oral biopsy	N/D	-	-

C3	S/D Oral biopsy	N/D	-	-

C4	S/D Oral biopsy	N/D	-	-

C5	S/D Oral biopsy	N/D	-	-

C6	S/D Oral biopsy	N/D	-	-

C7	S/D Oral biopsy	N/D	-	-

C8	S/D Oral biopsy	N/D	+	16

C9	S/D Oral biopsy	N/D	+	16

C10	S/D Oral biopsy	N/D	+	16

C11	S/D Oral biopsy	N/D	+	16

C12	S/D Oral biopsy	N/D	-	-

D1	S/D Oral biopsy	N/D	-	-

D2	S/D Oral biopsy	N/D	-	-

D3	S/D Oral biopsy	N/D	-	-

D4	S/D Oral biopsy	N/D	-	-

D5	S/D Oral biopsy	N/D	-	-

D6	S/D Oral biopsy	N/D	-	-

D7	S/D Oral biopsy	N/D	-	-

D8	S/D Oral biopsy	N/D	-	-

D9	S/D Oral biopsy	N/D	-	-

D10	S/D Oral biopsy	N/D	-	-

D11	S/D Oral biopsy	N/D	-	-

D12	S/D Oral biopsy	N/D	+	16

E1	S/D Oral biopsy	N/D	+	16

E2	S/D Oral biopsy	N/D	-	-

E3	S/D Oral biopsy	N/D	-	-

E4	S/D Oral biopsy	N/D	-	-

E5	S/D Oral biopsy	N/D	-	-

E6	S/D Oral biopsy	N/D	+	16

3	NS/ND Oral biopsy	16	N/D	16

4	NS/ND Oral biopsy	16	N/D	16

7	NS/ND Oral biopsy	-	N/D	-

9	NS/ND Oral biopsy	16	N/D	16

12	NS/ND Oral biopsy	16	N/D	-

13	NS/ND Oral biopsy	16	N/D	16

15	NS/ND Oral biopsy	-	N/D	-

16	NS/ND Oral biopsy	-	N/D	18

18	NS/ND Oral biopsy	-	N/D	-

20	NS/ND Oral biopsy	-	N/D	-

21	NS/ND Oral biopsy	18	N/D	-

23	NS/ND Oral biopsy	16	N/D	-

27	NS/ND Oral biopsy	-	N/D	-

32	NS/ND Oral biopsy	-	N/D	-

35	NS/ND Oral biopsy	18	N/D	6/18

36	NS/ND Oral biopsy	-	N/D	-

37	NS/ND Oral biopsy	18	N/D	-

38	NS/ND Oral biopsy	-	N/D	-

39	NS/ND Oral biopsy	18	N/D	-

40	NS/ND Oral biopsy	16	N/D	16

51	NS/ND Oral biopsy	16	N/D	16

52	NS/ND Oral biopsy	16	N/D	-

082481	Foot plantar wart	2	+	-

082458	Foot plantar wart	57	+	-

MX1		28	+	-

MX2		64	+	-

HPV6/11-related; *, HPV16-related; ‡, HPV18-related; §, N/D; not determined

### Design of specific primers and probes for multiplex detection of HPV types 6, 11, 16 and 18 by qPCR

**Figure 2:**
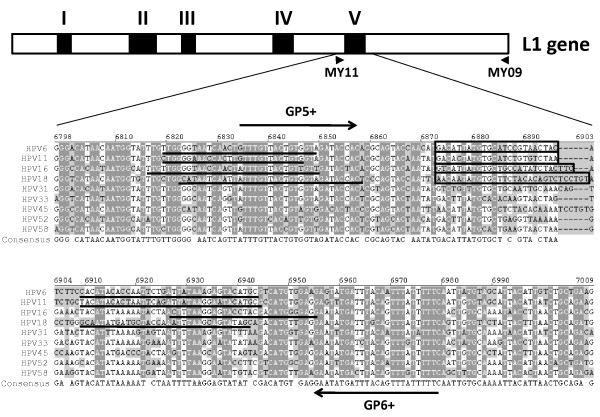
**Sequence and location of HPV type-specific L1 primers used for qPCR**. The top line represents a schematic diagram of the HPV L1 gene. Black boxes indicate the coding regions for the 5 hypervariable loop regions. The arrowheads indicate the position of the MY09/11primer pair used in traditional nested PCR. The coding region for the hypervariable loop V region is expanded below the schematic. Shown is an alignment of the HPV types identified in this study (HPV6, 11, 16 and 18) as well as other major oncogenic HPV types (HPV 31, 33, 45, 52, and 58). The type-specific primer sequences used for qPCR are underlined. Sequences corresponding to type-specific probes are boxed. Arrows indicate the position and sequence of the GP5+/GP6+ primer pair used in traditional nested PCR.

To amplify and detect the most commonly detected HPVs in mucosal disease type specific HPV, primers and TaqMan probes were generated corresponding to the L1 gene encoding the hypervariable V loop of the major capsid protein (Figure 2). This sequence falls within the region used for traditional PCR amplification with MY09/11 and/or GP5+/6+ primer pairs. Sequence alignment of the L1 gene with other oncogenic HPV types was performed to aid in the selection of primers and probes with maximum differences. Each HPV type-specific TaqMan probe was synthesized with a unique reporter dye at its 5' end. Fluorophores for reporter dyes were chosen that have the least overlap in their excitation/emission spectra to minimize background between detection channels.

### Derivation of standard curves for HPV type 6, 11, 16 and 18

**Figure 3:**
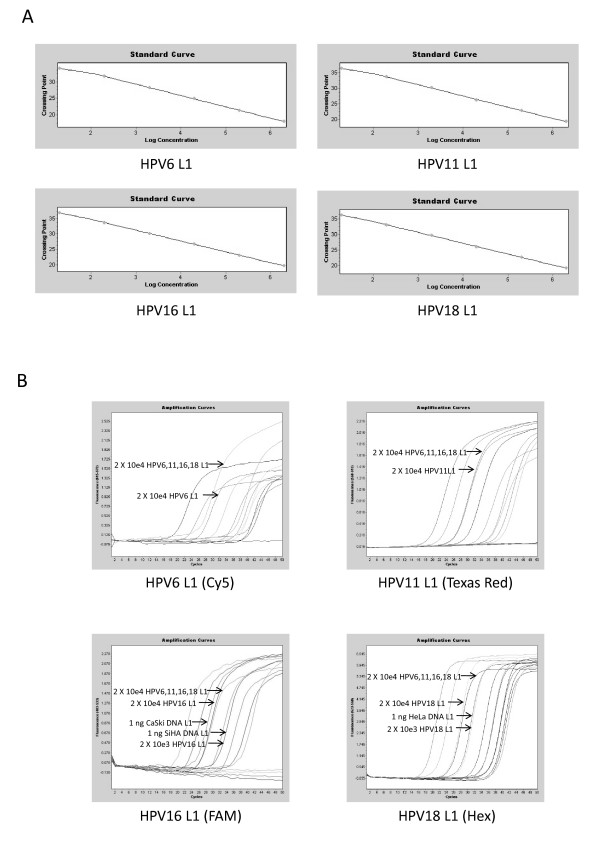
**Standard curve and amplification plots obtained for single qPCR assays**. (a) Reactions containing single type-specific primer pairs and probes were performed on a serial dilution of purified plasmids to generate standard curves that show a linear relationship between copy number and Ct value in a range from 2 × 10^1 ^to 2 × 10^6 ^copies/reaction. (b) Amplification plots for each excitation/emission spectrum are shown for each reaction containing a single type-specific primer pair and probe. Arrows indicate the amplification plots for reactions containing a mixture of each HPV type-specific L1 plasmid at 2 × 10^4 ^copies/reaction relative to the amplification plot corresponding to 2 × 10^4 ^copies for each type-specific HPV L1 standard. Arrows in the HPV 16 and 18 type-specific L1 reactions also indicate the amplification plots of CaSki (HPV16), SiHA (HPV16) and HeLa (HPV18) genomic DNA, respectively.

As an initial step in the development of a HPV type-specific quantitative assay, plasmids containing type-specific amplicons corresponding to the hypervariable V coding region of the L1 gene were used to generate standard curves in reactions containing a single HPV type primer pair and probe. Copy number was determined based on the size of each plasmid in nucleotide base pairs and calculated assuming a single deoxynucleotide base pair has a molecular weight of approximately 660 Daltons. Serial 10-fold dilutions of each plasmids ranging from 2 × 10^1 ^to 2 × 10^6 ^plasmids/μl were made. Initially, plasmids were used in qPCR assays containing a single HPV type-specific primer pair and probe and the Ct values were plotted against cycle number to determine limits of sensitivity. There was a linear relationship between the Ct values and cycle numbers when a range of between 2 × 10^1 ^copies and 2 × 10^6 ^copies of plasmid was present in the reaction, indicating that the assay was quantitative and had a lower limit of detection for each primer/probe combination of 20 copies/reaction (Figure 3A). These reactions were also spiked with a mixture containing equal copy numbers (2 × 10^4 ^copies) of each type-specific plasmids. In each type specific qPCR assay, primers and probes were able to distinguish and quantitate between their respective type-specific HPV (2 × 10^4 ^copies) and the other HPV types (6 × 10^4 ^copies) with minimal differences between the standard curve and the type-specific HPV present in the mixture (Figure 3B).

### Primer/probe combinations detect specific types of HPV L1 in multiplex reactions

**Figure 4:**
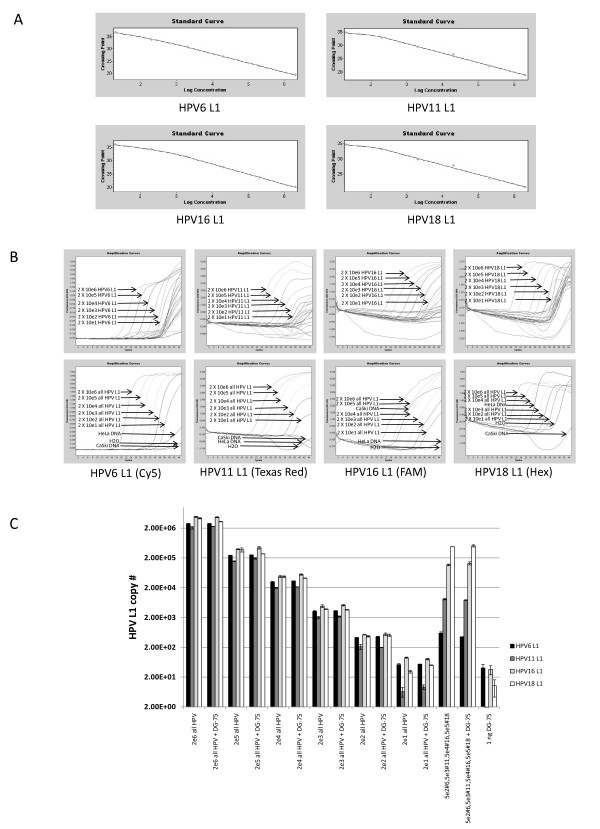
**The qPCR assay can specifically detect the HPV type L1 in a complex mixture containing all 4 L1 amplicons**. (a) Individual plasmids ranging from 2 × 10^1 ^to 2 × 10^6 ^copies were run in multiplex reaction mix to generate standard curves. (b) Multiplex reactions were performed on mixtures containing all 4 plasmids carrying type-specific amplicons. Mixtures contained equal amounts of each plasmid ranging in concentrations from 2 × 10^1 ^to 2 × 10^6 ^copies of each plasmid/reaction. The top panels show the standard curve amplification plots. The bottom panel shows the amplification plots for each plasmid mixture concentration range. CaSki and HeLa cell DNA were used as positive controls for HPV16 and 18, respectively. (c) Excess genomic DNA does not affect the performance of the multiplex HPV L1 qPCR assay. Reactions were performed using a mixture containing all 4 HPV L1 plasmids that ranged from 2 × 10^6 ^to 2 × 10^1 ^copies. Additional reactions were performed that contained a mixture of 5 × 10^5 ^copies of HPV6 L1, 5 × 10^4 ^copies of HPV11 L1, 5 × 10^3 ^copies of HPV16 L1 and 5 × 10^2 ^copies of HPV18 L1. The assay was performed in the presence or absence of 100 ng of DG-75 DNA to determine if excess human genomic DNA would affect the amplification and detection of type-specific HPV L1 DNA.

To determine if standard curves could be obtained in multiplex reactions, assays were performed on standard curve plasmids that contained all 4 primer pairs and probes. Linear plots of standards were similar in multiplex reactions compared to plots obtained when using single primer pairs and probes indicating that type-specific amplification of individual standard curves was unaffected by the presence of other type-specific primer pairs and probes (Figure 4A). To determine if primers and probes were specific for each type of HPV, reactions were spiked with a mixture of all four plasmids at 2 × 10^4 ^copies of HPV 6, 11, 16 and 18 L1 amplicons. While there was nonspecific detection of some plasmids at later times during the qPCR, the Ct values were well beyond the Ct values for the lowest concentration of each type-specific standard indicating that this detection would not interfere with the specific quantitation of each HPV type. If the multiplex qPCR was able to differentiate between HPV L1 types then there would not be significant deviation from the corresponding curve for copy number in each type-restricted amplification of the L1 gene. As was the case, addition of a mixture of HPV L1 amplicons did not affect the ability of each primer/probe combination to specifically amplify the corresponding type-specific L1 amplicon (Figure 4B). Multiplex qPCR was also performed on a single mixture of plasmids containing each of the L1 amplicon at a different concentration. There was good agreement in the quantitation of individual type-specific amplicons in the multiplex reactions and the predicted quantity of each amplicon added to the assay. This indicated that in a sample containing a mixture of four different HPV types the multiplex qPCR assay was able to specifically detect each type of HPV L1 gene.

In clinical samples an excess of cellular human DNA would be present in the reaction. To determine if cellular DNA would affect the specific amplification of each type of HPV, reactions containing a mixture of all four plasmids ranging from 2 × 10^6 ^to 2 × 10^1 ^copies of HPV 6, 11, 16 and 18 L1 amplicons were spiked with 100 ng of human genomic DNA obtained from the HPV-negative human B-cell line, DG-75. The presence of 100 ng of human cellular DNA did not affect the amplification of the target HPV L1 gene compared to unspiked controls in multiplex reactions (Figure 4C). This suggests that the presence of excess human cellular DNA would not affect the ability of the assay to specifically detect each type of HPV.

### Use of the novel HPV qPCR assays in clinical specimens

Overall 140 clinical specimens that originate from the oral cavity, skin and anogenital mucosa were assessed (Table 2). Both biopsies and fluids have been tested. A subset of specimens (anogenital/skin and oral cancers) have been assessed by both standard assays and by the novel assay.

#### Detection of HPV in well characterized anogenital and skin lesions

The ability of the 2 qPCR assays to detect HPV in clinical samples was assessed using DNA obtained from 35 well-characterized HPV-positive anogenital and skin samples. The integrity of cellular DNA was confirmed by analyzing Human Apolipoprotein B (HAPB) in each of the samples, the minimum level of HAPB was 10^3 ^copies. The multiplex qPCR assay specifically detected HPV6, 11, 16 or 18 in samples that had previously been shown to contain these types (Table 2). Sample 950496 was determined to have multiple HPV types present including HPV6 and HPV16 as determined by a nested PCR line blot assay. While this sample was positive for HPV6 by our multiplex qPCR assay it was negative for HPV16. The difference in the ability of these 2 assays to detected HPV16 in this sample may reflect the decreased sensitive of our qPCR assay due the single round amplification that is utilized. It should be noted that HPV13+ and HPV32+ have been detected in other clinical samples using the "broad spectrum" qPCR assay (unpublished results) suggesting that the inability of the assay to detect these 2 HPV types in their respective samples was due to insufficient viral loads despite having acceptable cellular HAPB levels.

#### HPV-type specific multiplex qPCR can detect and quantitate HPV in cervical lavage samples from HIV-positive individuals

DNA isolated from 25 cervical lavage samples obtained from HIV-positive women was used to determine if the multiplex qPCR assay could be used to detect the 4 most common HPV types in gynecological samples (Table 2) and to quantitate these viral loads (Table 3). Samples were deemed positive for HPV if their copy number was ≥10^3 ^as previously described [17]. HPV DNA was detected in 5/25 samples in the 6, 11, 16, 18 multiplex assay. Both HPV6 and HPV16 were detected in two of the 25 samples tested (Table 3). One sample was positive for both HPV6 and 18, another sample was positive for HPV18 only, and one sample was positive for HPV6 only. All of these samples were also positive for HPV using a Sybr Green-based qPCR assay that utilizes degenerative "broad spectrum" primers targeting the E1 gene, additionally 3 samples were detected that did not harbor HPV types 6,11,16 or 18. Thus 8/25 cervical samples were HPV-positive using the E1 broad spectrum assay (Table 2 and Table 3). These results are consistent with the finding that multiple HPV types are commonly found in cervical samples [23]. Additionally, three of the samples that were not positive for the 4 HPV types used in the multiplex assay were positive for HPV using the Sybr Green-based qPCR assay indicating that HPV types different than 6, 11, 16 and 18 were present in these samples. Sequence analysis of the EI products also detected types 35, 44, 67 and 68 (Table 3).

**Table 3:** Summary of multiplex HPV L1 qPCR and sybr green-based E1 qPCR assays.

		Multiplex TaqMan Reaction				Sybr Green Reaction	
**Specimen**	**Location**	**HPV6 L1**	**HPV11 L1**	**HPV16 L1**	**HPV18 L1**	**HPV E1**	**HPV E1 sequence**

CL2	Cervix	3.72E+05	0.00E+00	2.51E+04	0.00E+00	4.62E+06	mult HPV types

CL3	Cervix	1.00E+00	0.00E+00	1.00E+00	4.29E+04	2.31E+06	HPV44

CL6	Cervix	4.23E+05	0.00E+00	4.27E+04	0.00E+00	7.05E+06	HPV67

CL7	Cervix	6.30E+04	0.00E+00	1.00E+00	9.76E+03	5.10E+01	N/D

CL11	Cervix	1.00E+00	0.00E+00	1.00E+00	1.40E+01	4.73E+03	HPV35

CL18	Cervix	1.34E+02	0.00E+00	1.00E+00	4.00E+01	6.03E+04	mult HPV types

CL21	Cervix	3.27E+04	1.00E+00	2.82E+02	1.40E+01	2.11E+06	HPV68

CL23	Cervix	2.67E+02	4.00E+01	1.00E+00	4.00E+01	4.21E+04	mult HPV types

#### HPV-type specific multiplex qPCR can detect and quantitate HPV in oral cancers

Recent evidence has suggested that HPV plays a role in the development of oral cancers [5-7,24]. In contrast to cervical infections with HPV where multiple HPV types have been detected simultaneously, most oral infections involve a single type of HPV [23]. To determine whether HPV is associated with oral cancer, archived oral cancer tissue samples were obtained from the UNC Healthcare Cancer Registry. These samples were divided into two groups according to the patients smoking/drinking history. Because HPV associated cancers have a proclivity for distinct regions of the oral cavity, the location of the cancers is identified in Table 4. DNA obtained from the paraffin-embedded tissue was used in multiplex reactions to assess the ability of the qPCR assay to detect and quantitate HPV in clinical samples. Samples were considered positive for a specific HPV type if the copy number was ≥1000 copies [17].

**Table 4:** Anatomical location of oral cancer tissue used for the extraction of genomic DNA.

Smoker/Drinker oral cancer samples				Non-smoker/non-drinker oral cancer samples	
**Sample**	**Site**	**Sample**	**Site**	**Sample**	**Site**

A1	OC	C4	OC-gingiva	3	BOT

A2	HP	C5	larynx-TVC	4	BOT

A3	OC	C6	OC-RMT	7	BOT

A4	OC	C7	OC-gingiva	9	BOT

A5	OC	C8	OC-gingiva	12	BOT

A6	OC	C9	OP-BOT	13	BOT

A7	OC	C10	OP-tonsil	15	Tongue

A8	OC	C11	OC	16	Tongue

A9	OC	C12	larynx-supraglottis	18	Tonsil

A10	OC	D1	larynx-glottis	20	Tongue

A11	OC	D2	OP-tonsil	21	Tongue

A12	OC	D3	larynx-supraglottis	23	Tongue

B1	HP	D4	HP- post wall	27	Tongue

B2	Larynx	D5	larynx-epiglottis	32	Tongue

B3	OP-BOT	D6	OP-soft palate	35	Tonsil

B4	Larynx	D7	OC-tongue	36	Tonsil

B5	OC-tongue	D8	OC-tongue	37	Tonsil

B6	HP-pyriform	D9	HP-pyriform	38	Tonsil

B7	OC-RMT	D10	OP-tonsil	39	Tonsil

B8	OC-FOM	D11	OC-RMT	40	Tonsil

B9	OP-buccal	D12	OP-tonsil	51	BOT

B10	OC-gingiva	E1	HP-pyriform	52	BOT

B11	larynx-TVC	E2	OC-tongue		
		
B12	OP-BOT	E3	OP-FOM		
		
C1	OP-BOT	E4	OC-gingiva		
		
C2	OC-gingiva	E5	larynx-epiglottis		
		
C3	larynx-glottis	E6	OC-tongue		

BOT; base of tongue, FOM; floor of mouth, HP; hypopharynyx, OC; oral cavity, OP; oropharynyx, RMT; retromolar trigone, TVC; true vocal cord.

Initially, the multiplex qPCR assay was performed on 54 oral cancer samples from the population of patients with a history of smoking/drinking. Of these 54 samples, 8 (15%) had >1000 copies of HPV16/μg total DNA and were considered positive for HPV16 (Table 5).

**Table 5:** Similar HPV16 copy number are obtained from multiplex and individual HPV type-specific singleplex qPCR assays and universal E1 qPCR assay.

Sample	Site	HAPB	Multiplex HPV6 L1	Multiplex HPV11 L1	Multiplex HPV16 L1	Multiplex HPV18 L1	Singleplex HPV6 L1	Singleplex HPV11 L1	Singleplex HPV16 L1	Singleplex HPV18 L1	HPV E1
B12	OC	4.01e5	0	0	5.27e5	2.50e2	1.43e2	0	3.66e5	6.65e2	4.17e5

C9	HP	4.32e5	0	0	1.65e6	0	1.74e2	0	2.84e6	0	2.04e6

C10	OC	3.68e5	0	0	1.15e6	0	5.61e2	0	2.39e6	9.87e2	1.21e6

C11	OC	2.54e5	0	0	3.22e4	2.50e2	0	2.35e2	2.35e4	5.15e2	4.65e3

D12	OC	6.38e5	0	0	1.53e7	0	1.60e2	0	0.90e7	0	1.04e7

E1	OC	3.07e5	0	0	0.98e5	2.5e2	7.15e2	0	1.78e5	3.22e2	2.37e4

E6	OC	8.39e5	0	0	2.08e5	0	0	0	5.95e5	0	3.33e5

This percentage is consistent with other published reports of HPV detection in oral cancer in individuals who are smokers or drinkers [7]. None of the samples were positive for HPV6, 11 or 18. Type-specific singleplex reactions were performed on HPV16 positive samples to assess the specificity of the multiplex assay to identify specific HPV types (Table 5). All 7 HPV 16 multiplex positive samples were also positive for HPV16 DNA when assayed by the singleplex reactions. None of the samples were positive for HPV6, 11 or 18 in singleplex reactions indicating that specificity of the multiplex reaction and singleplex reaction were comparable. Detection of cellular HAPB in these samples reveal that they contained similar amounts of amplifiable cellular DNA. This sample set was also subjected to E1 broad spectrum PCR and only those that were positive for HPV 16 in the other assays were detected as positive. Unlike the cervical samples, other HPV types were not detected in these oral cancers. Similar HPV16 copy numbers were obtained from the multiplex reactions and E1 broad spectrum qPCR, compared to singleplex reactions indicating that both multiplex qPCR and E1 broad spectrum provided a reliable assessment of HPV16 copy number in these samples (Table 5).

**Figure 5:**
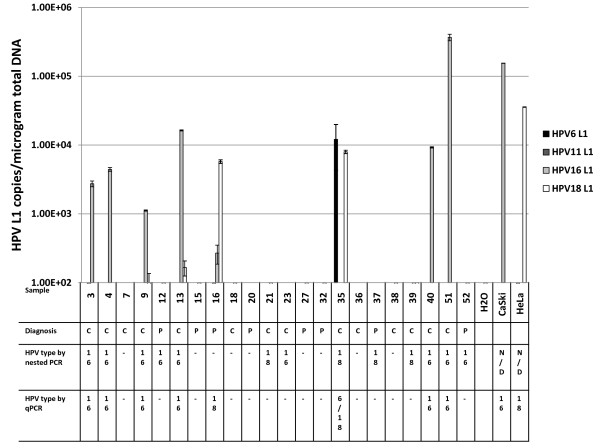
**Type-specific L1 DNA determined by multiplex qPCR correlates with typing determined by degenerative PCR using DNA from nonsmoker/nondrinker clinical samples**. DNA was extracted from paraffin-embedded tissue and used in the HPV type-specific L1 qPCR assay. C indicates the presence of cancer, P indicates that cancer was previously diagnosed and B indicates the tissue was histologically benign at the time biopsies were performed. The type-specific HPV L1 for each sample was previously determined by nested PCR using MY09/11 and GP5+/6+ primers pairs followed by direct sequencing of the PCR products. Homology of the PCR product with HPV16 is indicated by 16 while homology of the PCR product with HPV18 is indicated by 18. The inability to obtain a product by nested PCR is indicated by a dash. HPV type as determined by qPCR.

HPV is often detected in the oral cancers of non-smoker/non-drinker (NS/ND) patients [24]. To assess the performance of the multiplex qPCR assay to detect and differentiate between the 4 types of HPV, multiplex qPCR was performed using DNA obtained from oral cancers of non-smoker/non-drinkers that have previously been typed by sequencing products derived from degenerate PCR. These samples represent a cohort of non-smoker/non-drinker patients [24] who were either histologically diagnosed with head and neck cancer, or were at risk for recurrence (previous oral cancer/previously diagnosed). The site of the malignancy is detailed in Table 4. For follow up patients previously diagnosed with oral cancer a new biopsy at the site of the previous cancer was obtained. Samples were considered HPV-positive if their HPV copy number was above 1000 copies/μg of total DNA. Seven non-smoker/non-drinker cancer subjects were positive for HPV16 or 18 by multiplex qPCR. Six of the subjects (27%) tested positive for HPV16 while 2 subjects (9%) tested positive for HPV18 by multiplex qPCR (Figure 5). There was one subject who tested positive for both HPV6 and 18 by multiplex qPCR. Fifty seven percent of biopsies that were taken from invasive cancers (n = 14) were positive for either HPV16 or HPV18. Of the cancer biopsies 6 were positive for HPV16 while 2 were positive for HPV18. There were some discrepancies between HPV detection by nested PCR versus the multiplex qPCR assay. While 57% of cancers were positive using the multiplex analysis, 77% of cancers were positive using the nested PCR detection method. However, the 2 assays were run using DNA isolated from different sections of the same paraffin block. As viral distribution within the tissue may not have been homogeneous; this may account for differences in HPV detection by the 2 assays. Alternatively, the nested PCR while not quantitative may be inherently more sensitive due to second round of amplification during the nested PCR. Despite these discrepancies, there was reasonable concordance in the type-specific HPV identified in oral cancer biopsies by the qPCR assay compared with previous typing obtained by traditional MY9-MY11 L1 nested PCR followed by direct sequencing of the amplicons [24]. Type determination by the qPCR assay was concluded upon detection of a clear abundance of a specific type of HPV.

In summary, we **c**ompared our novel qPCR assay, which is a combination of E1 and L1 PCR results, to the gold standard method of "previously typed HPV" anogenital and oral cancer specimens (Table 2). Previously typed anogenital and skin specimens were typed using a standard PCR performed using MY09/MY11 consensus HPV L1 primers. Following PCR, specimens were typed by hybridizing to 29 different HPV types as well as 10 additional types together in a probe mixture as previously described [3]. In this instance, the concordance was absolute, leading to a sensitivity of 100% (91.0%, 100%). Oral cancer specimens, however were previously typed using nested PCR MY09/MY11 followed by GP5+/6+ and sequencing of the PCR product [24]. The sensitivity of the novel assays compared to this method was 53.9% (25.1, 80.1%). Specificity was determined by comparisons to true negatives as determined by the gold standard assays. It was determined that the specificity was 88.9%. Utilizing the data generated in Table 2, we detected an overall sensitivity of 89% (95% CI: 77% - 96%) and specificity of 90% (95%CI: 52% - 98%).

## Discussion

The causal relationship between oncogenic HPV and malignancy indicates the importance of being able to quickly detect and quantify specific HPV types when it is suspected that a lesion may harbor an HPV infection. This would aid in the assessment of precancerous lesions and impact on the decision to treat the lesion before it progressed to a more severe form of disease or allow the lesion to resolve on its own. In addition, low risk types of HPV also impact negatively on quality of life indicating the importance of tracking and identifying HPV types involved in the development of warts. Assays that are capable of distinguishing between common types of HPV responsible for cancers as well as those involved in the development of warts would facilitate the understanding of HPV epidemiology and pathology. In order to do so, two qPCR assays were developed for use in tandem. An E1 degenerate broad spectrum Sybr green-based assay was developed that would detect any HPV. In addition, the real-time qPCR that utilizes specific TaqMan probes labeled with different fluorophores represented an attractive method for quickly identifying and quantifying the presence of multiple viruses in a single sample, simultaneously, [25] and could be adapted to type-specific detection of HPV. Multiplexing such a reaction reduced the possibility of introducing errors inherent in performing multiple single type specific reactions for an individual sample, it also reduced the amount of time and reagents required to perform the assay. This would reduce the cost of performing the reaction thus making the assays more cost effective and increasing the accessibility of the assay to the application of clinical samples in general. While comparable L1 MY09/MY11 degenerate priming, these 1-step single tube qPCR assays have the additional advantage of being high throughput allowing for the detection of HPV in a a large number of samples in a relatively short amount of time when compared to the standard assay involving nested PCR followed by hybridization.

Assays were developed that detect HPV types 6, 11, 16 and 18; types targeted by the prophylactic quadrivalent vaccine. Each type-specific TaqMan probe contained a fluorophore that differed significantly in its excitation/emission spectra compared to other type-specific probes in the assay, and worked with equal efficiency allowing for the detection of multiple probes in a single reaction tube and reducing the time and reagents necessary to perform multiple individual reactions. The presence of large amounts of cellular genomic DNA did not affect the specific amplification and detection of amplicons. This assay had a linear range of detection over 6 orders of magnitude, allowing identification/typing of viral genomes and detection of viral load [11]. A recent study performed to determine HPV 6, 11, 16 and 18 seroprevalence in the US determined that many sexually active females become exposed to these HPV types, suggesting that continued evaluation is warranted to determine the impact of vaccination[26]. The qPCR assays described here may be instrumental in determining the efficacy of the quadrivalent HPV vaccine, particularly in vulnerable patients and will be instrumental in investigating HPV epidemiology in longitudinally collected samples. Increasing oncogenic HPV viral loads have been associated with development of malignancy [9]. While to our knowledge these associations have not yet been determined for other HPV associated non malignant conditions, it is reasonable to expect that viral loads precede disease development in that realm as well. These assays may be of significant use in deciphering the importance of HPV replication prior to disease development. The use of these qPCR assays have significant utility in distinguishing the presence of specific types of HPV. The one tube multiplex assay containing type/fluorophore specific TaqMan probes directed against HPV 6,11,16 and 18 provides a quick reliable method of distinguishing between HPV types and quantitating viral load while minimizing reagent costs and reducing errors.

In cases where other HPV types are involved, the "broad spectrum" HPV qPCR assay is instrumental in detection of HPV types that may be present but are not detected by the multiplex assay. While HPV16 and 18 have been shown to be responsible for the majority of HPV-related cancers, there are additional oncogenic types of HPV involved in malignancy. We used E1 degenerate primers in a sybr green-based qPCR assay that could amplify all HPV types. The E1 consensus region is approximately 150-200 bp, and is much more amenable to real time qPCR than the MY11-MY9 region which is 600 bp. To date, we are unaware of other primers sets used for HPV degenerate qPCR, however, the L1 based spf1/2 primers could serve as an alternative [27]. These primers generate a 65 bp fragment and have been used to detect 43 types of HPV in a hybridization line assay. The E1 assay is truly broad spectrum and was capable of detecting and quantitating 35 cutaneous and mucosal HPV types including HPV6, 11, 16 and 18 from previously characterized clinical specimens and provides an additional assay to augment the multiplex qPCR assay. Table 3 and 5 highlights the fidelity of these complimentary methods and clearly illustrates that in HPV 16 positive oral cancer samples, that were by multiplex were also positive by both singleplex and E1 broad spectrum assay. Further, similar levels of HPV 16 were detected by each of these methods. We have shown that we consistently detect the same range of HPV types as the standard MY09/11 and gp5+/6+. Theoretically we can detect more types because we are not limited to HPV types available on pre-blotted membranes used for hybridization.

The ability to detect and quantify specific HPV types in clinical samples allows for assessment of HPV-related disease. While used on cloned DNA/plasmids for types 6,11,16 and 18, the multiplex assay also performed optimally on well-validated anogenital and skin specimens. Our test shows both high sensitivity and specificity 89% and 90%, but it should be noted that the specificity estimate is based on only 9 true negatives, and thus, has a large confidence interval. Our sensitivity estimate's narrow confidence interval indicates that the true sensitivity of our test is likely higher than 77%. Takac et al [18] in their development of a realtime assay that detected 15 oncogenic and 5 low risk risk HPV types also had similar levels of sensitivity and specificity (95.45% and 91.57%, respectively). Among the clinical isolates there were HPV types 31, 33, 52,58, 67 and 35, not cross reactivity was detected with HPV 16. Likewise clinical isolates with HPV types 45, 59 did not cross react with HPV 18 nor did HPV 55 cross react with HPV 6/11 nor did HPV 6 cross react with 11 (Table 2, related types are indicated by symbols, Figure 4B and 4C). This highlights the specificity of the assay. There was nearly 100 percent agreement with regard to detection of HPV 6, 11, 16 and 18 and no cross reactivity with other types although there was disagreement in one of the specimens. Multiple HPV types including HPV 6 and 16 had previously been detected in sample 950496 using a nested PCR/line blot technique. While HPV 6 was detected by our multiplex assay, HPV16 was not detected. This discrepancy may be due to levels of HPV16 below the level required for detection by our qPCR assay. Compared to traditional nested PCR, the real time qPCR assays described here were able to distinguish between specific mucosal types of HPV and also had the added benefit of quantitating viral loads in cervical lavages and in head and neck tissue biopsied from a small cohort of oral cancer patients with high risk factors (smoking/drinking) as well as from non-smoker/non-drinker cancer patients. In the HIV-positive women approximately 31% of the cervical fluids were HPV-positive with multiple concurrent HPV infections. Interestingly, in the oral cancer group that had a history of smoking and/or drinking, only 15% of subjects were positive for HPV. In contrast oncogenic HPV could be detected in almost all of the cancer subjects in the non-smoking/non-drinking group suggesting that HPV plays a significant role in the development of oral cancers in the absence of additional risk factors. This is consistent with findings from this lab as well as others, showing low rates of oncogenic HPV infection in oral cancers of smoker/drinkers and high rates of infection in oral cancers obtained from non-smoker/non-drinkers [24,28].

With real time PCR technology becoming an increasingly common technology for clinical labs, this assay will allow for the rapid identification and quantification of pathogenic types of HPV. As viral replication often precedes the development of virus-associated pathology, the ability to quantitate HPV in persons 'at risk' for HPV associated disease may be an informative prognostic indicator. The novel qPCR assays described here may provide insight into the epidemiology and natural history of HPV infections within individuals and groups, providing prognostic capability and leading to the development of intervention strategies to prevent disease progression.

## Conclusions

We developed a single tube real time PCR assay capable of differentially detecting and quantifying 4 types of HPV represented in an FDA approved vaccine, types 6,11,16, and 18.

The ability of the assay to distinguish between these 4 types of HPV in a complex mixture of DNA and in the presence of human genomic DNA was validated. A "broad spectrum" sybr green-based qPCR assay that utilizes E1 degenerate primers that is capable of detecting multiple HPV types was also developed and validated. We showed that our assays performed as well as traditional nested PCR followed by sequencing/hybridization in distinguishing between different types of HPV in anogenital and oral fluids and tissues from high risk/diseased subjects.

## Abbreviations

HPV: Human papillomaviruses; HAPB: human apolipoproteinB; qPCR: quantitative PCR; ATCC: American Type Culture Collection

## Competing interests

The authors declare that they have no competing interests.

## Authors' contributions

WTS and JW-C prepared the manuscript and developed and performed the PCR assays. EA and MC identified and obtained oral cancer tissue. EMK and SC-U and identified and obtained cervical lavage samples from HIV infected individuals. JP identified and obtained well characterized HPV-positive anogenital samples used in validating the assays. AD performed the statistical analysis. All authors read and approved the final manuscript.
